# Novel Prodiginine Derivatives Demonstrate Bioactivities on Plants, Nematodes, and Fungi

**DOI:** 10.3389/fpls.2020.579807

**Published:** 2020-10-16

**Authors:** Samer S. Habash, Hannah U. C. Brass, Andreas S. Klein, David P. Klebl, Tim Moritz Weber, Thomas Classen, Jörg Pietruszka, Florian M. W. Grundler, A. Sylvia S. Schleker

**Affiliations:** ^1^INRES Molecular Phytomedicine, University of Bonn, Bonn, Germany; ^2^Institute of Bioorganic Chemistry, Heinrich Heine University Düsseldorf, Forschungszentrum Jülich, Stetternicher Forst, Jülich, Germany; ^3^IBG-1: Bioorganic Chemistry, Forschungszentrum Jülich GmbH, Jülich, Germany

**Keywords:** prodiginines, natural product, plant pathogens, plant protection, nematode

## Abstract

Bacterial metabolites represent an invaluable source of bioactive molecules which can be used as such or serve as chemical frameworks for developing new antimicrobial compounds for various applications including crop protection against pathogens. Prodiginines are tripyrrolic, red-colored compounds produced by many bacterial species. Recently, due to the use of chemical-, bio-, or mutasynthesis, a novel group of prodiginines was generated. In our study, we perform different assays to evaluate the effects of prodigiosin and five derivatives on nematodes and plant pathogenic fungi as well as on plant development. Our results showed that prodigiosin and the derivatives were active against the bacterial feeding nematode *Caenorhabditis elegans* in a concentration- and derivative-dependent manner while a direct effect on infective juveniles of the plant parasitic nematode *Heterodera schachtii* was observed for prodigiosin only. All compounds were found to be active against the plant pathogenic fungi *Phoma lingam* and *Sclerotinia sclerotiorum.* Efficacy varied depending on compound concentration and chemical structure. We observed that prodigiosin (**1**), the 12 ring- **9**, and hexenol **10** derivatives are neutral or even positive for growth of *Arabidopsis thaliana* depending on the applied compound concentration, whereas other derivatives appear to be suppressive. Our infection assays revealed that the total number of developed *H. schachtii* individuals on *A. thaliana* was decreased to 50% in the presence of compounds **1** or **9**. Furthermore, female nematodes and their associated syncytia were smaller in size. Prodiginines seem to indirectly inhibit *H. schachtii* parasitism of the plant. Further research is needed to elucidate their mode of action. Our results indicate that prodiginines are promising metabolites that have the potential to be developed into novel antinematodal and antifungal agents.

## Introduction

Plant pests (including insects, mites, plant parasitic nematodes), pathogens (e.g., viruses, bacteria, fungi), and weeds are major problems in crop production causing a share of the total global yield losses of up to 18%, 16%, and 34%, respectively (Oerke, 2006). In order to minimize crop losses, chemical, biological, and cultural means side by side with the use of resistant plants are management strategies in use (Bridge, 1996; Heydari and Pessarakli, 2010; Habash and Al-Banna, 2011; Timper, 2014). Each of these control means has its own challenges, but so far, pesticides are the fastest and the most effective combat means utilized. However, due to environmental hazards and toxicity to humans many pesticides are being banned. Therefore, effective and sustainable alternatives are needed.

**FIGURE 1:**
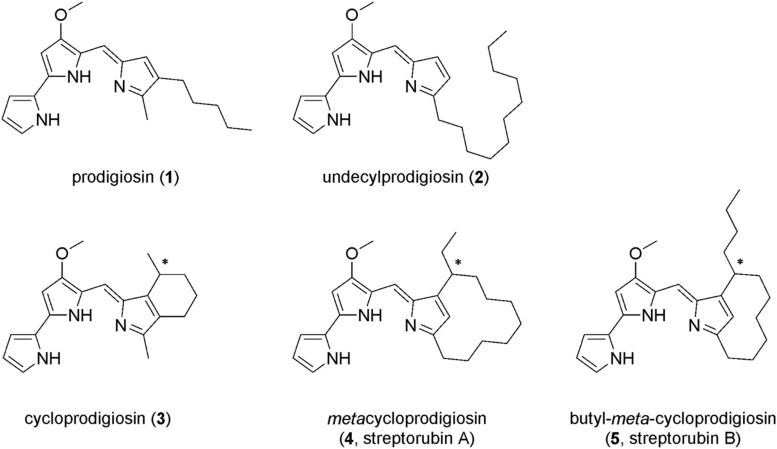
Examples of natural prodiginines: prodigiosin (**1**), undecylprodigiosin (**2**), cycloprodigiosin (**3**), *meta*cycloprodigiosin (**4**), and butyl-*meta*-cycloprodigiosin (**5**).

Microbial metabolites represent a valuable source of compounds that can be used for development of new drugs and plant protection agents. The bacterial prodiginines are nice examples with a wide range of bioactivities and intensively used in drug discovery research (Darshan and Manonmani, 2015). Prodiginines are tripyrrolic dark red compounds produced as secondary metabolites by many bacterial species within *Serratia* (Williams et al., 1956), *Streptomyces* (Gerber, 1975; Kawasaki et al., 2008), *Hahella* (Kim et al., 2007), and *Vibrio* (Starič et al., 2010), for instance. The family of prodiginines comprises diverse members (as shown in Figure 1), some having linear alkyl side chains such as prodigiosin (**1**) and undecylprodigiosin (**2**) while others have cyclic moieties such as cycloprodigiosin (**3**), *meta*cycloprodigiosin (**4**), or streptorubin B (**5**) (Williamson et al., 2006; Stankovic et al., 2014; Hu et al., 2016). In pharmaceutical studies, prodiginines were often used for their antimalarial and antitumor activities (Pérez-Tomás et al., 2003; Papireddy et al., 2011). They also exhibit inhibitory activities on various microbes. For example, *Escherichia coli* exhibited a leaky outer membrane and cells had severely decreased respiration activity upon exposure to prodigiosin (**1**) (Danevčič et al., 2016b). Likewise, treating *Pseudomonas aeruginosa* with prodigiosin (**1**), isolated from *Serratia marcescens*, altered the cell surface hydrophobicity, and biofilm integrity significantly. The treatment also caused nucleic acid degradation of *P. aeruginosa* (Kimyon et al., 2016). Recent research has shown potential applications of prodiginines in food industry as coloring agents, antioxidants, and antimicrobial additives to increase product shelf life (Arivizhivendhan et al., 2018).

In agricultural research, activities of prodigiosin (**1**) against several plant pathogens are described. It was shown that prodigiosin (**1**) totally inhibited spore germination of *Botrytis cinerea*, the causal agent of gray mold (Someya et al., 2001). The purified red pigment of *Serratia marcescens* was found to be effective against juvenile stages of the plant parasitic nematodes *Radopholus similis* and *Meloidogyne javanica* at low concentrations of 83 and 79 μg/mL, respectively, and inhibited nematode egg-hatching ability. After deep analysis of the bioactive red supernatant, prodigiosin (**1**) was detected in the supernatant (Rahul et al., 2014). This evidence supports the potential of the red pigment prodigiosin (**1**) to be applied in several fields.

The bio- and chemical-based derivatization of prodiginines introduces new compounds. These compounds could contribute to an improved microbe resistance management or having stronger bioactivities. Recently, nature inspired prodiginines were produced by combining organic syntheses with a mutasynthesis approach using the HV1-certified bacterium *Pseudomonas putida* KT2440 as host strain (Weist and Süssmuth, 2005; Kennedy, 2008; Kirschning and Hahn, 2012; Klein et al., 2017, 2018; Classen and Pietruszka, 2018). The previously constructed mutasynthesis strain harbors the prodigiosin (**1**) gene cluster from *S. marcescens*, but the bifurcated biosynthetic pathway is blocked upon gene deletion, thus, only one out of two prodiginine building blocks, namely 4-methoxy-2,2’-bipyrrole-5-carbaldehyde (MBC), is produced biosynthetically (Domröse et al., 2015; Klein et al., 2017). The other precursor and especially derivatives thereof are chemically synthesized and fed during cultivation, enabling the production of new and non-natural prodiginines (Klein et al., 2017, 2018). The obtained compounds possessed antibacterial activities against several species with minimum inhibitory concentrations (MIC) ranging from 0.1 to 12 μMM. In the same studies, they demonstrated that the produced prodiginines exhibit modulating activities of autophagy in human breast cancer cells. The novel compounds with such bioactivities could be good candidates to be used against plant pathogens. Their attraction is increased by the fact that prodiginines act on microorganisms in a mode different compared to conventional pesticides.

In our study we present the bioactivities of prodigiosin (**1**), several cyclic derivatives **6**–**9** and a hydroxylated prodiginine **10** against different organisms, particularly focusing on the plant parasitic nematode *Heterodera schachtii*. First, we examined the bioactivities of prodiginines on two nematode species belonging to bacterial feeding and plant parasitic nematodes. We further investigated the effect of the compounds on plant growth using the model plant *A. thaliana* and subsequently evaluated the efficacy of selected compounds in combating nematode parasitism in the pathosystem *A. thaliana–H. schachtii*. Finally, we tested the bioactivities of prodiginines on other pathogens by using two species of plant pathogenic fungi.

## Materials and Methods

### Prodiginine Production

#### Production of Prodiginines **1**, **6–9** *via* Biosynthesis or Mutasynthesis

**FIGURE 2:**
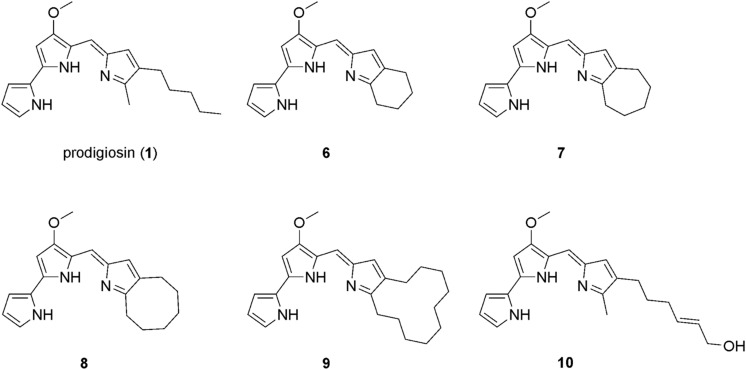
Chemical structure of prodigiosin (**1**) and the derivatives **6–10** that were investigated in this study.

Prodigiosin (**1**) was produced by using the strain *P. putida* pig-r2 as previously described (Domröse et al., 2015). The prodiginine derivatives **6**–**9** (Figure 2) were produced *via* mutasynthesis as previously described (Klein et al., 2017, 2018). For details, see Supplementary Material.

#### Chemical Synthesis of Prodiginines

Synthesis of **10** is described in detail in Supplementary Material.

### Nematode Culture

The wild type of the bacterial feeding nematode *C. elegans*, used in this work, was maintained on nematode growth medium (NGM) and was fed with *E. coli* strain OP50. In the toxicity assay the synchronized first stage nematodes were used (Al Banna et al., 2018). The plant parasitic nematode *H. schachtii* Schmidt used in the experiments was reared on white mustard (*Sinapis alba* L. cv. Albatros) plants which were grown aseptically on 0.2% Knop agar medium. Mature cysts were collected in funnels and hatched in 3 mM ZnCl_2_ (Sijmons et al., 1991). The hatched pre-parasitic J2s were collected and used in the experiments.

### Effect of Prodigiosin Derivatives on Nematodes

To investigate the effect of prodigiosin derivatives on nematode motility, two nematode species were used. Both nematodes, *C. elegans* and *H. schachtii*, were challenged with the selected compounds **1**, **6**–**10**. The activity experiment was conducted in 96-well plates (Greiner Bio-One) under aseptic conditions. Each well contained 90–100 nematodes incubated in 60 μL of the compound-containing test solution. The used dilutions were 100, 50, 25, 12.5, and 6.25 μM dissolved in 0.5% (v/v) dimethyl sulfoxide (DMSO) diluted in ddH_2_O. Nematodes soaked in 0.5% (v/v) DMSO served as control. Plates were incubated for two days at 24°C in the dark. Numbers of active (moving) and inactive (not moving) nematodes were evaluated using a dissecting microscope. Nematode movement was provoked by adding 2 μL of 1 M sodium hydroxide (NaOH). Those nematodes that remained immobile after challenging with NaOH were regarded as dead. The percentages of nematode motility were calculated. The experiment was set up in three biological replications (each contains three wells per concentration). To decide whether *H. schachtii* J2s were really killed or just paralyzed by prodigiosin (**1**), J2s were washed free of the compound after a 48 h incubation period and subsequently left in water for another 48 h. This was necessary as some *H. schachtii* J2s incubated in **1** showed a slight movement of the tail region after adding NaOH what made a reliable decision difficult. Data were collected and statistically analyzed by one-way analysis of variance (ANOVA) followed by Dunnett’s post-hoc test to determine significant differences to the control (SigmaPlot 12.5).

### Plant Growth Test

In order to investigate the effect of prodiginines on plants, *A. thaliana* ecotype Col-0 was grown aseptically on agar medium supplemented with modified 0.2% Knop solutions at 16 h light and 8 h dark at 25°C as described previously (Sijmons et al., 1991). Five-days-old plants were transferred to 6-well plates containing 2 mL liquid Murashige and Skoog medium (MS) supplemented with the prodiginines **1**, **6**–**10** at different concentrations (50, 25, 12.5 μM), MS medium supplemented with 1 μM bacterial flagellin (flg22) or with 0.5% (v/v) DMSO were used as controls. Fresh weight of the roots and the shoots were measured after 15 days of incubation. The experiments were performed in five technical replicates and independently repeated three times. Data were statistically analyzed using one-way ANOVA followed by Dunnett’s post-hoc test.

### Infection Assay

In order to investigate the impact of prodigiosin derivatives on nematode parasitism, prodigiosin (**1**) and prodiginine **9** were selected to be tested. The compounds and the used concentration were chosen based on the previous results of the plant development assay. Compounds were added to the 0.2% Knop agar medium to yield a final concentration of 14 μM. *A. thaliana* Col-0 plants were grown aseptically on the medium with 16 h light and 8 h dark at 25°C as described previously (Sijmons et al., 1991). The infection assay with *A. thaliana* plants was performed as described previously (Habash et al., 2017). Briefly, roots of 10 days old seedlings were inoculated with 60–70 *H. schachtii* J2s per plant. Twelve days after inoculation (DAI), numbers of adult males and females were counted per plant. Furthermore, sizes of females and associated syncytia were measured after 13 DAI using Leica M165C Binocular (Leica Microsystems, Wetzlar, Germany) and Leica Application Suite software. *A. thaliana* plants grown on 0.2% Knop agar medium supplemented with 0.5% (v/v) DMSO were used as control. Experiments were repeated three times and statistically analyzed using one-way ANOVA followed by Dunn’s post-hoc test. Each experiment consisted of 12 plants per treatment.

### Effect of Prodigiosin Derivatives on Plant Pathogenic Fungi

The plant pathogenic fungi *Phoma lingam* and *Sclerotinia sclerotiorum* were used in this study to evaluate the bioactivity of the compounds on hyphal growth. Both isolates were obtained from the Leibniz-Institut DSMZ (Deutsche Sammlung von Mikroorganismen und Zellkulturen GmbH, Germany) and subcultured on potato dextrose agar (PDA) at 24°C. To test prodiginine bioactivities, PDA was supplemented with the chemicals to several final dilutions. The used concentrations were 50, 25 and 12.5 μM. Fungal discs were cut from the culture media and placed in the middle of PDA plates containing the chemicals. PDA plates with 0.5% (v/v) DMSO alone were used as control. All plates were incubated for 7 days at 24°C. Subsequently, the diameter of the fungal colony was measured, and inhibition percentage was calculated. Experiments were repeated four times, each experiment consisted of 3 technical replicates. Differences between the treatments were statistically analyzed by using one-way ANOVA followed by Dunnett’s post-hoc.

## Results

### Activity of Prodiginines Against Nematodes Is Dependent on Compound Structure and Concentration as Well as Nematode Species

**FIGURE 3:**
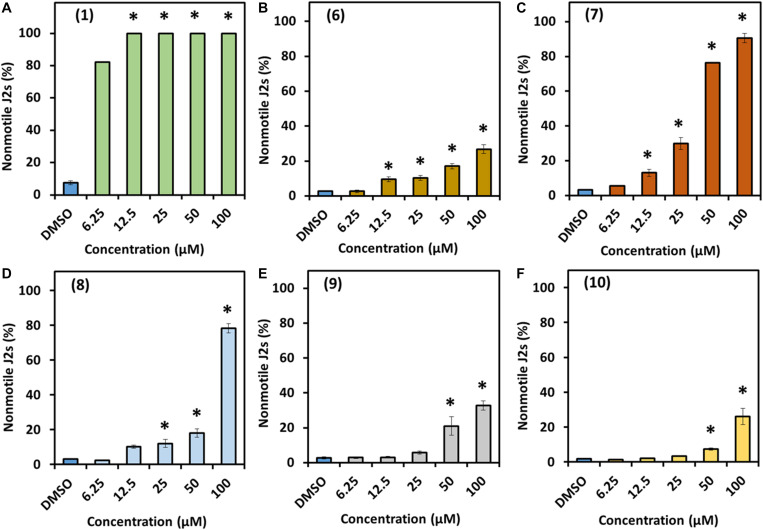
Direct effect of prodigiosin (**1**) and derivatives **6–10** on *C. elegans* first-stage juveniles. **(A)** Compound 1, **(B)** compound 6, **(C)** compound 7, **(D)** compound 8, **(E)** compound 9, and **(F)** compound 10. Values are means ± standard error of three biological replicates (*n* = 9). Asterisks indicate significant differences to the control based on one-way ANOVA followed by Dunnett’s post-hoc test (*P* < 0.05).

The bioactivity of prodigiosin (**1**) and the derivatives **6**–**10** was determined using two representative soil nematodes with different feeding behavior, the bacterial feeding model nematode *C. elegans* and the plant parasitic nematode *H. schachtii.* The two tested nematode species responded differently to exposure to prodiginines. Results revealed that the impact of the tested compounds on *C. elegans* depends on prodiginine structure and concentration (Figure 3). Prodigiosin (**1**) showed the highest nematicidal activity against *C. elegans* juveniles with 0.127 μM being the effective concentration that killed half of the treated nematodes (EC_50_). This was followed by **7** (35.9 μM), **8** (79.1 μM), and then the other three derivatives. For compounds **10**, **6**, and **9** a reliable EC_50_ could not be determined as only 26, 27, and 33% effectivity, respectively, could be observed at the highest test concentration.

**FIGURE 4:**
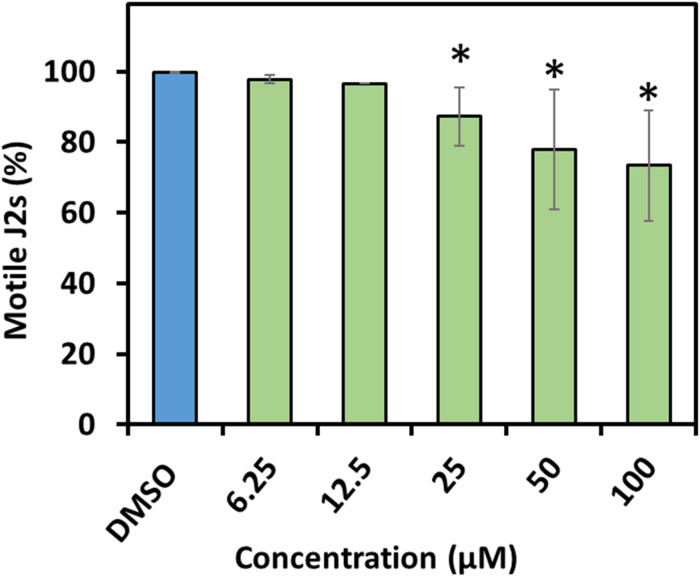
Recovery of second stage juveniles of *H. schachtii* after exposure to prodigiosin (**1**). Percentage of recovered nematodes after 48 h incubation in prodigiosin (**1**) followed by washing and incubation for 48 h in water. Values are means ± standard error of three biological replicates (*n* = 9). Asterisks indicate significant differences to the control based on one-way ANOVA followed by Dunnett’s post-hoc test (*P* < 0.05).

The compounds were much less effective against *H. schachtii* J2s and rather had a static effect. In fact, prodigiosin (**1**) was the only derivative able to cause considerable paralysis of the second stage juveniles. The calculated 48 h EC_50_ value was 13.3 μM and the J2s were able to recover even from the 100 μM treatment as only 27% of the J2s were observed to be dead after a 48 h recovery phase. The impact of a 48 h exposure of the J2s to any other derivative only had a very low neglectable effect with a maximum of about 10% immobile nematodes (100 μM) thus not enabling us to calculate the EC_50_ (Table 1 and Figure 4).

**TABLE 1:** Direct effect of prodigiosin (**1**) and derivatives **6–10** on *H. schachtii* second-stage juveniles.

Compound	Concentration (μm)	Average nonmotile J2s (%)	SE
(**1**)	DMSO	4.4	0.7
	6.25	29.9*	2.4
	12.5	52.0*	2.8
	25	67.2*	3.4
	50	87.2*	1.6
	100	93.3*	1.5
(**6**)	DMSO	1.0	0.7
	6.25	2.8	0.8
	12.5	2.8	0.1
	25	1.4	0.2
	50	1.3	0.1
	100	9.3*	1.3
(**7**)	DMSO	2.2	1.0
	6.25	2.7	0.6
	12.5	2.0	0.7
	25	2.0	0.3
	50	2.3	0.4
	100	10.4*	1.3
(**8**)	DMSO	1.2	1.0
	6.25	2.1	0.6
	12.5	1.8	0.9
	25	1.8	0.5
	50	1.4	0.3
	100	6.0*	1.9
(**9**)	DMSO	1.4	0.4
	6.25	1.9	0.4
	12.5	1.8	0.7
	25	1.7	0.8
	50	2.2	1.1
	100	5.4*	0.5
(**10**)	DMSO	1.0	0.9
	6.25	1.3	0.3
	12.5	1.6	0.8
	25	2.0	1.2
	50	5.2*	2.8
	100	10.0*	4.7

*Values are means ± standard error of three biological replicates (n = 9). **^∗^**Indicates significant differences to the control based on one-way ANOVA followed by Dunnett’s post-hoc test (P < 0.05).*

### Effect of Prodigiosin (**1**) and Derivatives **6–10** on Plant Growth

**FIGURE 5:**
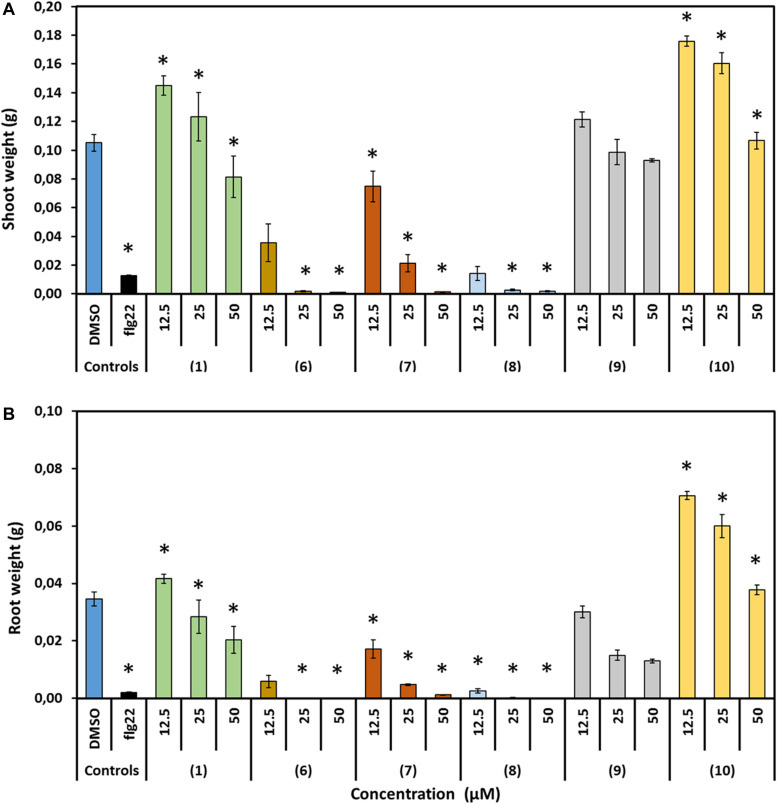
Effect of prodigiosin (**1**) and derivatives **6–10** on *A. thaliana* growth. Figures show growth parameters representing development of *A. thaliana* cultivated on media mixed with prodigiosin (**1**) or different derivatives (**6**–**10**) in three concentrations compared with those growing on medium mixed with DMSO as control. Flg22 (1 μM) treated plants served as controls for successful growth inhibition. **(A)** Average fresh shoot weight (g). **(B)** Average fresh root weight (g). Data are based on three independent experiments. Each bar represents the mean ± standard error of *n* = 15. Asterisks indicate significant differences to the control based on one-way ANOVA followed by Dunnett’s post-hoc test (*P* < 0.05).

The effect of prodiginines on plant growth was tested in order to obtain a suitable concentration for the subsequent infection assay and to get an indication which compound could be best suited for a possible use as plant protection agent in agricultural applications. Therefore, shoot weight and root weight were determined. The applied concentrations between 12.5 and 50 μM of the derivatives **6** and **8** inhibited plant growth as shoot weight was decreased by more than 70% compared to the DMSO control. Compound **7** was also detrimental for the plant but less severe. Cultivating the plants in 25 and 50 μM significantly decreased plant shoot weight, whereas the impact of 12.5 μM was not significant compared to the DMSO control. On the other hand, incubating plants in prodigiosin (**1**, 12.5 μM) and derivative **10** (12.5 and 25 μM) increased shoot weight by up to 70%. Higher concentrations of both derivatives had no significant effect. All concentrations of **9** had no significant effect on shoot weight compared with the control (Figure 5).

Comparable effects of the compounds on root weight could be observed. Compound **10** was highly promoting root growth at concentrations of 25 or 12.5 μM. Prodigiosin (**1**) treatment was neutral except for the 50 μM concentration were root weight was significantly reduced. Root weight was decreased significantly when plants were incubated in 50 μM of all other derivatives. The effect was less severe when the concentration was decreased to 25 or 12.5 μM but still root weight was significantly decreased compared to the control with the exception of **9**, where this inhibitory effect was demolished when plants were incubated in 12.5 μM.

### Prodiginines Inhibit Nematode Parasitism

**FIGURE 6:**
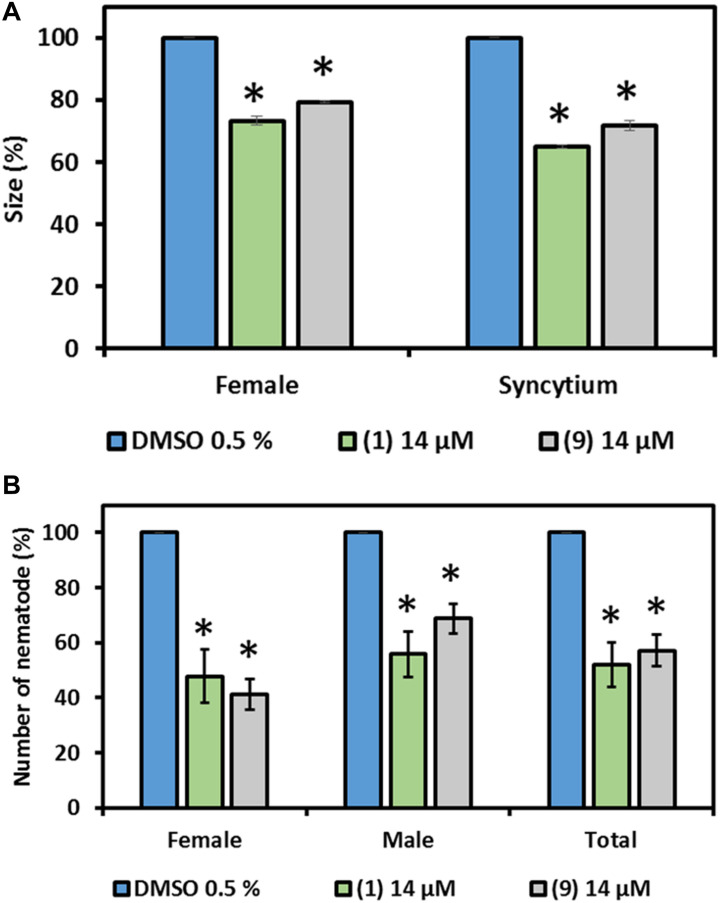
Effect of prodigiosin (**1**) and derivative **9** on *H. schachtii* parasitism of *A. thaliana*. Plants were grown on Knoṕs agar mixed with prodigiosin (**1**) and derivative **9** and were infected with J2s of *H. schachtii*. Susceptibility parameters were **(A)** average percentage of females, males and total nematodes per plant compared with control and **(B)** average percentage of sizes of females and syncytia at 13 DAI. Plants grown on medium mixed with DMSO (0.5% final concentration) were used as control. Data are based on three independent experiments. Each bar represents the mean ± standard error of *n* > 35. Asterisks indicate significant differences to the control based on one-way ANOVA followed by Dunnett’s post-hoc test (*P* < 0.05).

Due to the observed neutral to positive effect of prodiginines on plant growth and compound availability, **1** and **9** were selected to investigate their effect on *H. schachtii* parasitism of *A. thaliana*. A final concentration of 14 μM of each molecule was incorporated into the plant growth medium. Medium mixed with DMSO in a final concentration of 0.5% was used as control. Results showed that the number of developed females and males was significantly decreased on the plant roots in presence of compound **1** or **9** compared with the control (Figure 6). The number of females was decreased by up to 60%, while the males were up to 42% less in the treatments compared to the control. In total, a reduction of up to 50% in total infection was observed when plant roots are exposed to prodigiosin (**1**) or the derivative **9**.

Furthermore, the size of developed females and their associated syncytia were measured in order to investigate nematode development. Both, developed females and their associated syncytia were smaller in size compared with the control in the presence of compound **1** or **9** in the medium. The female size reached 77 and 80% of that of the females of the control in presence of prodigiosin (**1**) and **9**, respectively. The inhibitory effect on syncytium development was higher as the average size determined was 60 and 75% of the size in the control in presence of prodigiosin (**1**) and **9**, respectively (Figure 6).

### Bioactivities Against Plant Pathogenic Fungi

**FIGURE 7:**
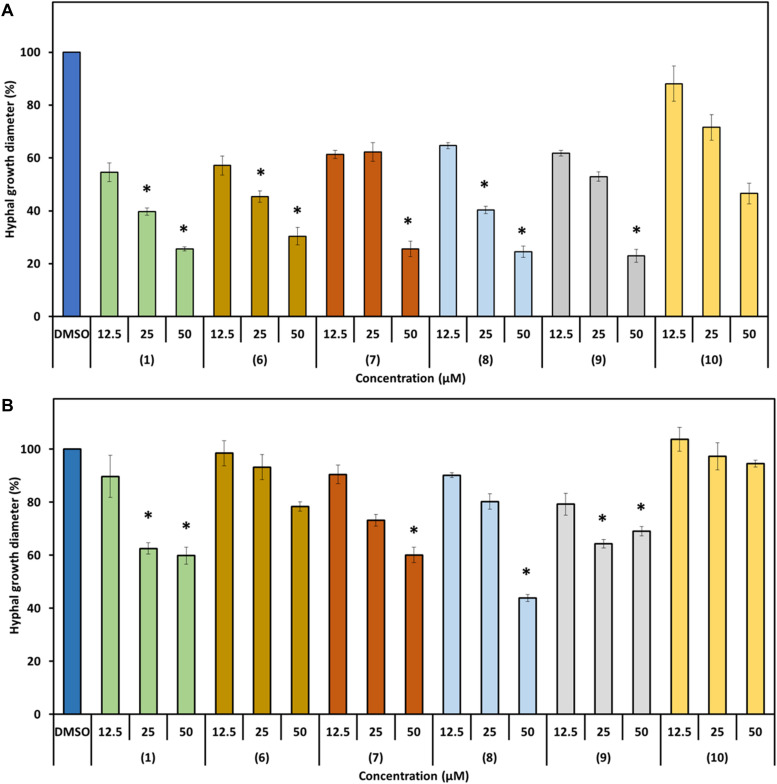
Effect of prodigiosin (**1**) and derivatives **6–10** on fungal hyphal growth. Figures show percentage of hyphal growth of the plant pathogenic fungi cultivated on media mixed with prodigiosin (**1**) or the derivatives **6**–**10** in three concentrations compared with those cultivated on medium mixed with DMSO as control. **(A)** *P. lingam*. **(B)** *S. sclerotiorum*. Values are means ± standard error of four biological replicates (*n* = 12). Asterisks indicate significant differences to the control based on one-way ANOVA followed by Dunnett’s post-hoc test (*P* < 0.05).

We further investigated the bioactivity of prodiginines against the two plant pathogenic fungi *P. lingam* and *S. sclerotiorum.* Both fungi share host plants with *H. schachtii*. Besides observing a possible antifungal activity, we aimed at getting an indication whether the changes in bioactivity documented for the structurally different prodiginines against nematodes follow a similar pattern in their activity against fungi. The two fungi responded differently to direct compound exposure and the degree of inhibition of hyphal growth varied with the prodiginine and concentration used (Figure 7). All tested derivatives except **10** significantly inhibited the hyphal growth of *P. lingam* at 50 μm. When the fungus was cultured on medium containing 25 μM of the compounds, prodigiosin (**1**), **6** and **8** showed significant inhibition. While none of the used compound showed inhibitory activity on hyphal growth at 12.5 μM.

*S. sclerotiorum* was less sensitive toward the used prodiginines. All compounds inhibited hyphal growth except derivatives **6** and **10,** even when 50 μM was used (Figure 7B). Fungal growth varied depending on the tripyrrol applied and ranged from 44% (**8**) to 100% (**10)** compared to the control (Figure 6B).

## Discussion

Management of plant pathogens in agricultural crop production is indispensable, no matter whether organic, integrated or conventional farming practices are applied. In particular, control of plant parasitic nematodes is extremely challenging. Rising public health concerns about pesticides harmful for the environment led to several effective compounds being banned from the market. This in return, limited the pathogen management options of the farmer during production processes. These reasons urge to find environmentally safe and sustainable alternatives to control pathogens and ensure yield and food quality.

Naturally occurring bacterial metabolites represent a valuable source of active compounds that can be used in different applications including pathogen control. In the last decade, several studies investigated these potential compounds (Schrader et al., 2010; Pathma et al., 2011; Zhai et al., 2018). Prodigiosin (**1**) which is a tripyrrolic red-colored pigment produced by many bacterial species got attention due to its bioactivities and was intensively studied (Darshan and Manonmani, 2015; Yip et al., 2019). Derivatization of compounds is one of the important tools to increase the availability as well as the activity of compounds. Various structure activity studies have already proven that variation of the prodiginine structure leads to different biological activities (D’Alessio et al., 2000; Papireddy et al., 2011; Marchal et al., 2014a, b; Kancharla et al., 2015). Synthetic biology is one of the approaches that helps this process and provides the tools necessary for applying mutasynthesis or biotransformation concepts for a controlled production of prodiginines in a safe heterologous bacterial host (Klein et al., 2017, 2018). Also biocatalysis approaches employing only the final enzyme of the prodigiosin (**1**) gene cluster and chemically synthesized precursors can lead to new prodiginine structures (Brass et al., 2019).

In our current study, we introduce several lines of results showing the potential of prodigiosin (**1**) and several derivatives including a new prodiginine with a terminal hydroxy group adjacent to an allylic double bond and their bioactivities toward several organisms including nematodes, fungi and plant. We first tested all compounds against the model nematode *C. elegans* and the plant parasitic nematode *H. schachtii* in direct exposure assays. The nematode *C. elegans* is frequently used in studies involved in investigating compounds’ bioactivities against multicellular organisms (Sun et al., 2017; Al Banna et al., 2018; Liao, 2018). The nematode *H. schachtii* is one of the devastating pathogens of several economically valuable crops (Steele, 1965; Muller, 1999). We observed that all derivatives are nematicidal for *C. elegans* while basically only the lead structure prodigiosin (**1**) is static for *H. schachtii* J2s. The nematicidal activity of the compounds toward *C. elegans* is clearly dependent on the molecule structure and the concentration with compounds **6**, **9**, and **10** having the lowest efficacy. The difference in compound activity toward the two nematode species could be due to differences in feeding habit. *C. elegance* has a wider mouth part which is an open canal that allows to uptake compounds from the environment while in *H. schachtii* the uptake through the mouth is controlled by the spear-like stylet and feeding exclusively occurs at the host plant during the sedentary phase. Thus, the mobile infective juvenile stage of *H. schachtii* used here is not feeding at all consequently limiting uptake of compounds from the environment to passive processes. Same results were demonstrated when both, *C. elegans* and *Meloidogyne incognita*, were exposed to the nanoparticles of silicon carbide. *C. elegans* was much more sensitive and exhibited high mortality as well as accumulation of the nanoparticles in the body after the exposure, while very low mortality and particle accumulation were observed in case of *M. incognita* (Al Banna et al., 2018). Furthermore, the two related nematodes, *H. schachtii* and *Globodera rostochiensis*, behaved differently upon exposure to exogenous application of several amino acids. In these studies, *G. rostochiensis* was shown to be sensitive to the application of methionine while no effect was observed in case of *H. schachtii* (Evans and Trudgill, 1971; Blümel et al., 2018). Both evidences support the hypothesis that different nematode anatomy and biology affect the nematodes’ sensitivity toward compounds. The nematicidal activities of prodigiosin (**1**) itself was also observed previously. It was shown that the survival of juvenile stages of *Radopholus similis* and *Meloidogyne javanica* was affected after treatment with the red pigment extracted from *S. marcescens* with LC_50_ (lethal concentration) value of 83 and 79 μg/mL, for *R. similis* and *M. javanica*, respectively. After deep analysis of the pigment using several liquid chromatography approaches and spectroscopic analysis, they confirmed the presence of prodigiosin as a bioactive metabolite (Rahul et al., 2014). All these findings support our results and show that different nematode species could behave differently due to exposer to compounds.

In order to introduce such compounds as agrochemicals, the effect on the plant has to be tested. No previous work was done studying the effect of prodiginines on plant development. Here, we report the effect of prodigiosin (**1**) and derivatives on plant growth of the model plant *A. thaliana* for the first time. *Arabidopsis* growth was different between treatments and was depending on concentration and prodiginine structure. We observed that prodigiosin (**1**) and the 12-ring derivative **9** are neutral for plant growth when low concentrations are applied. In case of the prodiginine **10**, plant growth was even highly promoted at low concentrations.

Based on the plant growth experiment results and compound availability, prodigiosin (**1**) and the 12-ring derivative (**9**) were chosen and a proper concentration was defined to be used to test the effect of the molecules on nematode parasitism on *A. thaliana*. The presence of 14 μM of both compounds in the plant growth medium affected *H. schachtii* parasitism and decreased the total number of adult nematodes per plant up to 50%. Furthermore, the compounds affected nematode development which is reflected by the smaller developed females and associated syncytia. Interestingly, although prodigiosin (**1**) paralyzes the J2s in liquid medium, we could not observe an impact of prodigiosin-supplemented agar on J2 motility. Therefore, we suggest that the observed effect of the two compounds on *H. schachtii* parasitism could be explained by compound-triggered plant defense responses. Similar observations were reported when no direct effect was detected after incubating J2s of *H. schachtii* in amino acids but still decreased parasitism when amino acids were integrated in the growth medium (Blümel et al., 2018). The logical explanation of such an outcome is that the presence of the compounds in the growth medium is activating plant defense responses against the nematode. These tests should be complemented by soil-based experiments in the future to confirm the observed control potential under field conditions. Particularly because in natural environments, factors like compound leaching, soil properties and compound stability affect efficacy.

We tested the bioactivities of the prodiginines against two plant pathogenic fungi. Both fungi can infect the same host plants as *H. schachtii*, thus a double impact of the compounds would increase their value. Our results showed that the direct effect of the prodiginines against the fungi *P. lingam* and *S. sclerotiorum* was even stronger compared to nematodes. The hyphal growth of both tested fungi was affected by the presence of prodigiosin (**1**) and derivatives **6***–***10** in the growth medium. Earlier, it was shown that prodigiosin (**1**) is active against several fungi and oomycetes. The purified prodigiosin (**1**) from *S. marcescens* F-1-1 inhibited the germination of cystospores and the growth of hyphae of *Phytophthora capsici*. It also appeared to be active against the plant pathogenic fungi *Cochliobolus miyabeanus*, *Pythium spinosum* and *P. ultimum* (Okamoto et al., 1998). According to our results, hyphal growth of *P. lingam* was affected more than that of *S. sclerotiorum*. Such a difference is dependent on fungal species and also demonstrated by previous studies. Chen et al. (2014) presented that the novel fungicide 3-[5-(4-chlorophenyl)-2,3-dimethyl-3-isoxazolidinyl] pyridine (SYP-Z048) affected several pathogenic fungi and the effect was variable between the tested fungi.

Comparing the results of the assays, we show that prodigiosin (**1**) is the most effective and derivative **10** is the least effective obviously reflecting the variability in compound sensitivity between different organisms. Particularly, *P. lingam* appears to be very sensitive to all compounds whereas *H. schachtii* only responded to prodigiosin (**1**). Interestingly, the compounds’ impact on plant development does not completely follow the same trend as seen for nematodes and fungi. Although plant growth promotion is highest for the prodiginine with the overall lowest activity against nematodes and fungi (compound **10**), prodigiosin (**1**) is neutral for plant growth depending on the concentration applied.

Compound **9** is a very good example that structural modification of a bioactive lead structure can guide compound activity toward desired properties. The lead structure **1** and derivative **9** have a comparable impact on plant development (neutral) and on parasitism of *H. schachtii* on *A. thaliana* (inhibition). Intriguingly, **1** is highly toxic for *C. elegans*, which is a representative of non-target organisms, whereas **9** does not affect this nematode’s vitality at concentrations suitable to control *H. schachtii* and *P. lingam*, which are two important pathogens on sugar beet.

Further detailed investigations are needed to unravel the mode of action against nematodes. Although studies with nematodes are missing so far, mechanistic insights of the effect of prodigiosin in other organisms were reported. For example, it was shown that the motility of the bacteria *Bacillus cereus*, *Staphylococcus aureus*, *Pseudomonas aeruginosa*, and *Escherichia coli* was inhibited upon exposure to prodigiosin. The inhibition was combined with activation of several features of programmed cell death (Darshan and Manonmani, 2015). In another study, the exposure of *B. subtilis* to prodigiosin was associated with growth inhibition and cell membrane leakage. The authors suggested that prodigiosin affects the bacterial cell by inducing cell lysis activities (Danevčič et al., 2016a). Similarly, *E. coli* exhibited a leaky outer membrane and cells had severely decreased respiration activity upon exposure to prodigiosin (Danevčič et al., 2016b).

Our current results demonstrate the bioactivities of prodigiosin (**1**) and its derivatives (**6**–**10**) against plant pathogenic fungi and parasitic nematodes and thereby introduce new natural compounds that have the potential to be developed into novel plant protection agents.

## Data Availability Statement

All datasets presented in this study are included in the article/Supplementary Material.

## Author Contributions

AS, FG, JP, TC, and SH conceived the research concept and designed the experiments. SH performed the assays on plants, nematodes, and fungi. HB, AK, DK, and TW produced and synthesized the used prodiginines. SH drafted the manuscript with input from all authors. All authors reviewed and approved the final manuscript.

## Conflict of Interest

The study was the basis for a patent application. Patent applicant: Forschungszentrum Juelich GmbH; inventors: SH, HB, AK, DK, TW, TC, JP, FG, and AS application number: 10 2020 116 516; status: examination phase.
