# Supplementary material for: Novel Prodiginine Derivatives Demonstrate Bioactivities on Plants, Nematodes, and Fungi
Source: Front Plant Sci. 2020 Oct 16;11:579807. doi: 10.3389/fpls.2020.579807 (PMC7596250; doi:10.3389/fpls.2020.579807)
Supplement: Supplementary file 1 [file Data_Sheet_1.docx]

Supplementary Material

# Supplementary Methods

## Biological production of prodiginines

### Biosynthesis of prodigiosin

Prodigiosin (**1**) was produced by using the strain *Pseudomonas putida* pig‑r2 as previously described (Domröse et al., 2015). A 3 L Fernbach flask with 500 mL containing TB (terrific broth) liquid medium (Carl Roth, Karlsruhe, Germany: 12 g/L casein, 24 g/L yeast extract, 12.54 g/L K_2_HPO_4_, 2.31 g/L KH_2_PO_4_, 4 mL/L glycerol) was inoculated to an OD_600_ of 0.02 with an overnight culture of *P. putida* pig‑r2. 5 g Polyurethane (PU) foam cubes (Bornewasser, Göllheim, Germany: Softpur, 25 kg/m^3^ density, 4 kPa compression hardness), each approx. 1 cm^3^, were added to the medium for in situ extraction of prodigiosin (**1**). The culture was incubated at 25 °C for 24 h. The foam cubes were recovered from the cell culture by sieving, then wrung out, washed with water and afterwards extracted with diethyl ether (300 mL) under usage of a 250 mL Soxhlet apparatus with a reflux condenser and a 500 mL round bottom flask. After extraction, the organic layer was washed with water, dried over MgSO_4_ and the solvent was removed under reduced pressure providing the desired crude extract of **1**, which was further purified via column chromatography on silica gel with dichloromethane + MeOH [gradient: 0.5–2% (*v*/*v*)]. Precipitation as hydrochloride provided the prodigiosin (**1**) as a dark red solid (41–53 mg/L).

### Production via mutasynthesis

The prodiginine derivatives **6**–**9** were produced via mutasynthesis as previously described (Klein et al., 2017, 2018). The synthesis of the pyrrole precursors was performed as described in Klein et al. (Klein et al., 2017, 2018). The mutasynthesis reactions were performed in 3 L Fernbach flasks using a final volume of 500 mL containing TB liquid medium, 0.5 mm pyrrole precursor and 2% (*v*/*v*) DMSO. 5 g Polyurethane (PU) foam cubes, each approx. 1 cm^3^, were added to the medium as an adsorbent for in situ extraction of prodiginines. The TB liquid medium was inoculated with an overnight culture of 4‑Methoxy‑2,2′‑bipyrrol‑5‑carbaldehyd (**15**, MBC) producing *P. putida* pig‑r2 Δ*pigD* (Klein et al., 2017) to an OD_600_ of 0.02 and Streptomycin was added as antibiotic to the culture in a final concentration of 80 µg/mL. The pyrrole precursors (**11**–**14**) were dissolved in DMSO beforehand (62.5 mm) and added to the culture medium to achieve the final concentration. After the addition of the PU foam cubes the mutasynthesis proceeded at 25 °C and 125 rpm for 24 h. The recovery and extraction process of the foam cubes was performed as described above. The crude extracts of **7**–**9** were purified via column chromatography on silica gel with dichloromethane + MeOH [gradient: 0.5–2% (*v*/*v*)]. Precipitation as hydrochloride provided the prodiginines as a dark red solid (**6**: 3.1 mg/L, **7**: 1.2 mg/L, **8**: 3.0 mg/L, **9**: 0.6 mg/L) (**Supplementary Figure S1**).





**Supplementary Figure S1.** Mutasynthesis approach for the production of prodiginine derivatives **6**–**9**.

## Chemical syntheses and analytical data

### General methods

Names are in accordance with the IUPAC nomenclature.

**General Experimental Considerations:** All chemicals being used were purchased from the companies Sigma-Aldrich/Fluka, TCI International, Alfa Aesar and VWR International/Merck. Pure solvents were either purchased or distilled prior to use. Absolute solvents were either taken from a drying machine (*MBraun*, model MB SPS-800), or distilled. The pH value of the buffer was adjusted using a pH-meter 766 Calimatic^®^. Thin layer chromatography (TLC) was conducted on POLYGRAM^®^ SIL G/UV254 plates with fluorescence indicator. Detection was either by UV absorption or treatment with KMnO_4_ or *p*-anisaldehyde solution followed by heating. Preparative column chromatography was performed using silica gel 60, particle size 0.04‒0.063 mm (230‒240 mesh) or neutral aluminum oxide 90.

**Mass spectrometry:** GC-MS analysis was performed on a Thermo Scientific Trace 1310 gas chromatograph (*Thermo* *Scientific,* Waltham, MA, USA) equipped with a Optima 5MS column (30 m x 0.25 mm, 0.25 μm, *Macherey-Nagel*, Düren, Germany) and coupled with a ISQ™ QD Single Quadrupole Mass Spectrometer (*Thermo* *Scientific,* Waltham, MA, USA). The temperatures of the injector and the detector were fixed at 250 °C and 230 °C, respectively. Helium was used as the carrier gas. Mass spectra were collected in the electron impact mode at 70 eV. The column temperature was initially 60 °C for 1 min, then raised to 185 °C at a rate of 15 °C min^−1^, subsequently raised to 280 °C at a rate of 120 °C min^−1^ and maintained at that temperature for 5 min.

**NMR spectroscopy:** ^1^H‑ and ^13^C‑NMR spectra were recorded on an Advance/DRX 600 nuclear magnetic resonance spectrometer (*Bruker,* Billerica, USA) at ambient temperature in CDCl_3_ at 600 and 151 MHz, respectively. The chemical shifts are given in ppm relative to tetramethylsilane [^1^H: δ(SiMe_4_) = 0.00 ppm] as an internal standard or relative to the solvent [^1^H: δ(CDCl_3_) = 7.26 ppm; ^13^C: δ(CDCl_3_) = 77.16 ppm]. Signals were assigned by means of DEPT-135° Pulse-, ^1^H-^1^H-COSY-, ^1^H-^13^C-HSQC- und ^1^H-^13^C-HMBC-experiments; splitting patterns are given as singlet (s), doublet (d), triplet (t), quartet (q), multiplet (m) and broad singlet (brs) plus coupling constants (*J*) are reported in Hz.

**IR spectroscopy**: IR data were recorded on a *SpectrumTwo* instrument (*PerkinElmer,* Waltham, MA, USA) as thin film. Absorbance frequencies are reported in cm^-1^.

**LC-MS (achiral stationary phase):** Analytes were separated and analyzed using a LC-MS *Agilent* 1100 series (*Agilent Technologies*, Santa Clara, CA, USA) equipped with a diode array and API electrospray mass detector. Substances were separated by the reversed phase stationary phase Atlantis T3 (1 m x 3.0 mm, 3 µm). Water + 0.1% (*v*/*v*) formic acid and methanol + 0.1% (*v*/*v*) formic acid were used as eluents for the following gradient program: 0.00 min: water + 0.1% (*v*/*v*) formic acid : methanol + 0.1% (*v*/*v*) formic acid (90:10), 4.00 min: water + 0.1% (*v*/*v*) formic acid : methanol + 0.1% (*v*/*v*) formic acid (40:60), 6.00 min: 100% methanol + 0.1% (*v*/*v*) formic acid. The program was stopped after 10.00 min. Flow rate was set to 0.6 mL/min. The column temperature was kept at 30 °C. Detection wavelengths were 510 nm, 520 nm, 530 nm, 540 nm, 3D field (190 nm–800 nm). 10 µL of each sample were injected. MS detection was set to positive mode with a range of *m*/*z* = 100–1000. Substances were identified by their UV absorption spectra and their mass to charge ratio (*m*/*z*).

### Syntheses of prodiginines 9 and 10

**Synthesis of 9**

Prodiginine synthesis of **9** was performed following the method previously described (Brass et al., 2019). The reaction was performed in a *Schlenk* flask under nitrogen atmosphere and Boc‑MBC (**16**, 92.6 mg, 0.32 mmol, 1.00 eq.) and 4,5,6,7,8,9,10,11,12,13-decahydro-1*H*-cyclododeca[*b*]pyrrole (**14**, 98.3 mg, 0.48 mmol, 1.50 eq.) were dissolved in 8 mL dry methanol. A 1.25 m solution of HCl in methanol (332 µL, 0.42 mmol, 1.30 eq.) was added at 0 °C, and the solution was stirred for 7 h. Upon addition of HCl in methanol, the solution turned dark red immediately. The solution was stirred for 15 h at room temperature until the reaction was stopped by addition of a few drops ammonia solution [25% (*w*/*w*)] at 0 °C whereby the reaction mixture turned from red to orange. The reaction mixture was extracted with dichloromethane (3 × 25 mL). The combined organic layers were dried over MgSO_4_ and the solvent was removed under reduced pressure. The crude extract of **9** was purified via column chromatography [silica, dichloromethane/ ammonia in methanol (7 n) (0.5‑2% (*v*/*v*) gradient), followed by chromatography on neutral aluminum oxide, *n*‑pentane/EtOAc (gradient: 95:5, 50:50, 0:100)]. Precipitation as hydrochloride provided **9** (58.3 mg, 0.14 mmol, 44%) as a dark red solid (**Supplementary Figure S2**).





**Supplementary Figure S2.** Synthesis of prodiginine derivative **9**.

**Synthesis of 10**

Prodiginine synthesis of **10** was performed as follows. 4-Methoxy-5-{[5-methyl-4-(pent-4-en-1-yl)-2*H*-pyrrol-2-ylidene]methyl}-1*H*,1′*H*-2,2′-bipyrrol∙HCl (**18**) was synthesized beforehand from Boc‑MBC (**16**) and 2‑methyl‑3‑(pent‑4‑en‑1‑yl)‑1*H*‑pyrrole (**17**) as described above. To a solution of 4‑methoxy‑5‑{[5‑methyl‑4‑(pent‑4‑en‑1‑yl)-2*H‑*pyrrol‑2‑ylidene]methyl}-1*H*,1′*H-*2,2′‑bipyrrol∙HCl (**18**, 50.0 mg, 140 µmol, 1.00 eq.) and *cis*‑2‑buten-1,4‑diol (46 µL, 0.56 mmol, 4.0 eq.) in dry dichloromethane (5 mL) *Hoveyda‑Grubbs Catalyst*™ 2^nd^ Generation Catalyst (9 mg, 10 mol‑%) was added at 0 °C. The reaction mixture was stirred for 18 h at room temperature. Afterwards the solvent was evaporated and the crude extract purified *via* column chromatography [silica, dichloromethane, + Gradient 0.5–4% (*v/v*) 7 n NH_3_ in MeOH]. Precipitation as hydrochloride provided **10** as a dark red solid (18 mg, 46 µmol, 33%) (**Supplementary Figure S3**).





**Supplementary Figure S3.** Synthesis of prodiginine derivative **10**.

### Compound characterization

For compound characterization of prodiginines **1**, **7**–**9** please refer to (Domröse et al., 2015; Klein et al., 2018). For compound **10** is as follow:

**(*E*)-6-(2-{[4-Methoxy-1*H*,1′*H*-(2,2′-bipyrrol)-5-yl]methylen}-5-methyl-2*H*-pyrrol-4-yl)hex-2-en-1-ol∙HCl (10)**

**^1^H‑NMR** (600 MHz, CDCl_3_): δ [ppm] = 1.64 (tt, ^3^*J*_7″,6″_ = 7.6 Hz, ^3^*J*_7″,8″_ = 7.6 Hz, 2H, 7″‑H), 2.09 (dt, ^3^*J*_8″,7″_ = 7.1 Hz, ^3^*J*_8″,9″_ = 7.1 Hz, 2H, 8″‑H), 2.41 (t, ^3^*J*_6″,7″_ = 7.6 Hz, 2H, 6″‑H), 2.53 (s, 3H, 13″‑H), 4.00 (s, 3H, 7‑H), 4.11 (d, ^3^*J*_11″,10″_ = 5.2 Hz, 2H, 11″‑H), 5.68 (m_c_, 2H, 9″-, 10″‑H), 6.08 (s, 1H, 3‑H), 6.35 (m_c_, 1H, 4′‑H), 6.66 (s, 1H, 3″‑H), 6.92 (dd, ^3^*J*_3′,4′_ = 4.0 Hz, ^4^*J*_3′,5′_ = 2.0 Hz, 1H, 3′‑H), 6.93 (s, 1H, 8‑H), 7.22 (s, 1H, 5′‑H), 12.55 (brs, 1H, 1′‑NH), 12.69 (brs, 2H, 1-, 1″‑NH); **^13^C‑NMR** (151 MHz, CDCl_3_): δ [ppm] = 12.4 (C‑13″), 24.8 (C‑6″), 29.5 (C‑7″), 31.7 (C‑8″), 58.8 (C‑7), 63.7 (C‑11″), 92.9 (C‑3), 111.8 (C‑4′), 115.9 (C‑3′), 117.3 (C‑8), 120.9 (C‑5), 122.2 (C‑2′), 125.1 (C‑2″), 127.1 (C‑5′), 127.7 (C‑4″), 128.2 (C‑3″), 129.6 (C‑9″), 132.4 (C‑10″), 146.6 (C‑5″), 147.9 (C‑2), 165.9 (C‑4); **IR** (ATR‑Film): $\tilde{\nu}$ [1/cm] = 3161, 2919, 1631, 1603, 1573, 1544, 1511, 1409, 1358, 1261, 1135, 1119, 1067, 1040, 993, 961, 837, 753; **HRMS** (ESI-FTMS, positive-ion): calculated for C_21_H_26_N_3_O_2_ (M + H)^+^ = 352.20189, found = 352.20195; **LC‑MS** (API‑ES, 70 eV): t_R_ = 6.50 min (**Supplementary Figure S4**).





**Supplementary Figure S4.** Structure of prodiginine derivative **10**.

### Analytical data





**Supplementary Figure S5.** ^1^H‐ and ^13^C‐NMR‐spectra of **10** in CDCl_3_ (600 MHz/151 MHz).

## Supplementary References

Brass, H. U. C., Klein, A. S., Nyholt, S., Classen, T., and Pietruszka, J. (2019). Condensing Enzymes from *Pseudoalteromonadaceae* for Prodiginine Synthesis. Adv. Synth. Catal. 361, 2659-2667. doi:10.1002/adsc.201900183.

Domröse, A., Klein, A. S., Hage-Hülsmann, J., Thies, S., Svensson, V., Classen, T., et al. (2015). Efficient recombinant production of prodigiosin in *Pseudomonas putida*. Front. Microbiol. 6, 972. doi:10.3389/fmicb.2015.00972.

Klein, A. S., Brass, H. U. C., Klebl, D. P., Classen, T., Loeschcke, A., Drepper, T., et al. (2018). Preparation of cyclic prodiginines by mutasynthesis in *Pseudomonas putida* kt2440. ChemBioChem 19, 1545–1552. doi:10.1002/cbic.201800154.

Klein, A. S., Domröse, A., Bongen, P., Brass, H. U. C., Classen, T., Loeschcke, A., et al. (2017). New Prodigiosin Derivatives Obtained by Mutasynthesis in *Pseudomonas putida*. ACS Synth. Biol. 6, 1757–1765. doi:10.1021/acssynbio.7b00099.
